# Embedding Group VIII Elements into a 2D Rigid pc-C_3_N_2_ Monolayer to Achieve Single-Atom Catalysts with Excellent OER Activity: A DFT Theoretical Study

**DOI:** 10.3390/molecules28010254

**Published:** 2022-12-28

**Authors:** Qingxian Wang, E Yang, Ran Liu, Mingyue Lv, Wei Zhang, Guangtao Yu, Wei Chen

**Affiliations:** 1 Engineering Research Center of Industrial Biocatalysis, Fujian Provincial Key Laboratory of Advanced Materials Oriented Chemical Engineering, Fujian-Taiwan Science and Technology Cooperation Base of Biomedical Materials and Tissue Engineering, College of Chemistry and Materials Science, Fujian Normal University, Fuzhou 350007, China; 2 Academy of Carbon Neutrality of Fujian Normal University, Fuzhou 350007, China; 3 Fujian Provincial Key Laboratory of Quantum Manipulation and New Energy Materials, College of Physics and Energy, Fujian Normal University, Fuzhou 350117, China; 4 Fujian Provincial Key Laboratory of Theoretical and Computational Chemistry, Xiamen University, Xiamen 361005, China

**Keywords:** single-atom catalyst (SAC), oxygen evolution reaction (OER), electrocatalyst, 2D pc-C_3_N_2_ monolayer, DFT calculations

## Abstract

Under DFT calculations, a systematic investigation is carried out to explore the structures and oxygen evolution reaction (OER) catalytic activities of a series of 2D single-atom catalyst (SAC) systems, which are constructed by doping the transition metal (TM) atoms in group VIII into the cavities of rigid phthalocyanine carbide (pc-C_3_N_2_). We can find that when Co, Rh, Ir and Ru atoms are doped in the small or large cavities of a pc-C_3_N_2_ monolayer, they can be used as high-activity centers of OER. All these four new TM@C_3_N_2_ nanostructures can exhibit very low overpotential values in the range of 0.33~0.48 V, even smaller than the state-of-the-art IrO_2_ (0.56 V), which indicates considerably high OER catalytic activity. In particular, the Rh@C_3_N_2_ system can show the best OER performance, given that doped Rh atoms can uniformly serve as high-OER-active centers, regardless of the size of cavity. In addition, a detailed mechanism analysis was carried out. It is found that in these doped pc-C_3_N_2_ systems, the number of outer electrons, the periodic number of doped TM atoms and the size of the embedded cavity can be considered the key factors affecting the OER catalytic activity, and excellent OER catalytic performance can be achieved through their effective cooperation. These fascinating findings can be advantageous for realizing low-cost and high-performance SAC catalysts for OER in the near future.

## 1. Introduction

Since the 21st century, the environmental and energy crisis has become increasingly serious. Developing new clean energy sources can be regarded as an effective strategy to alleviate this major problem [1,2]. Some obvious advantages of hydrogen energy, such as rich reserves and the lack of pollution to the environment after combustion, make it one of the most promising energy carriers [3,4]. The electrocatalytic splitting of the most abundant water on the earth has been considered to be the most efficient and sustainable hydrogen production method [5,6]. It is well known that the oxygen evolution reaction (OER) at the anode, as one half-reaction of water splitting, involves a complex four-electron transfer process, and it is kinetically slow and is usually the rate-determining step [7,8]. Both IrO_2_ and RuO_2_ materials are recognized as the most efficient OER catalysts [9,10]. However, they cannot be widely used due to their high cost and low abundance. Therefore, it is urgent to find some alternative, efficient and sustainable OER catalyst.

Different strategies have been proposed to design efficient OER catalysts, mainly by reducing the content of precious metals and developing nonprecious metal alternatives [11,12,13,14,15,16,17,18,19]. With the development of synthetic technologies, the emergence of single-atom catalytic materials has promising prospects for achieving nonprecious and highly active OER catalysts. Compared with the traditional catalysts, single-atom catalysts (SACs) have shown great potential advantages, including high selectivity [20,21], tunable high activity [22] and maximum atomic efficiency [23]. At present, more and more attention has been paid to the design of efficient SAC catalysts for OER. For example, Zhang et al. found that inserting a Ni atom into a defect-rich graphene can achieve an SAC catalyst with good OER activity [24]. Lin et al. also reported that a SAC catalyst with high OER activity can be synthesized by anchoring Pt atoms on a metal oxide NiO nanotube [25]. Moreover, Chen et al. explored the OER catalytic activity of a series of monoatomic transition metals supported by the MXenes M_2_NO_2_ (M = Ti, V and Cr) through first principle calculations and found that a Cu-anchored Ti_2_NO_2_ system can be used as an efficient SAC catalyst for OER [26]. Obviously, some different supports have been used to construct high-efficiency SAC catalysts, and the selection of appropriate support can play a crucial role in determining the high OER catalytic activity.

Different from the previous investigations, in this study, we mainly focus on a two-dimensional (2D) pc-C_3_N_2_ monolayer containing two different cavities [27], which are surrounded by four N atoms in pyrrole-like units or four N atoms connecting the pyrrole-like units. This kind of unique porous structure with two different sizes of holes can be advantageous for trapping TM atoms with different atomic radii to form two different series of TMN_4_ centers in pc-C_3_N_2_, exhibiting promising OER catalytic activity. Besides, the high stability and excellent conductivity of a pc-C_3_N_2_ monolayer [28] can also be conducive to its development as a suitable support for constructing potential SAC structures.

In this work, the TM atoms (TM = Fe, Co, Ni, Ru, Rh, Pd, Os, Ir and Pt) in group VIII are embedded into the cavities of a fascinating carbon–nitrogen substrate pc-C_3_N_2_ to construct a series of new TM@pc-C_3_N_2_ structures with TMN_4_ centers. It is highly anticipated that some of these structures can exhibit considerably high OER catalytic performance. Herein, we have carried out systematic first-principles calculations to investigate their structures, electronic properties and OER catalytic activities. The following issues will be mainly addressed: (i) Does the introduction of these TM atoms of group VIII enhance the conductivity of these systems? (ii) Which TM atoms can be embedded to form SAC catalysts with high OER activity? (iii) How do the number of outer electrons and the periodic number of TM atoms affect the OER catalytic activity? (iv) Does the hole size have any effect on the OER catalytic performance? The solution of these problems will facilitate the design of new SAC catalysts with high OER catalytic performance based on the relevant systems with rigid framework, including pc-C_3_N_2_. 

It is worth mentioning that our calculation results reveal that a series of new 2D structures with high stability and excellent conductivity can be obtained by doping these group VIII TM atoms into a porous pc-C_3_N_2_ monolayer. Among them, pc-C_3_N_2_ systems doped with Co, Rh, Ir and Ru atoms can uniformly exhibit a very low overpotential value in the range of 0.33~0.48 V, even smaller than the state-of-the-art IrO_2_ (0.56 V), which indicates the considerably high OER catalytic activity. This is the result of the combined effect of the number of outer electrons, the periodic number of doped TM atoms and the size of the embedded cavity.

## 2. Calculation Method

All the DFT calculations were performed by using the Vienna Ab initio Simulation Package (VASP) code [29]. The projector augmented wave (PAW) method [30,31,32] was used to describe the ion–electron interaction. The electronic exchange-correlation was approximated by the Perdew–Burke–Ernzerhof (PBE) functional in the generalized gradient approximation (GGA) [33], which includes the semiempirical van der Waals (vdW) method proposed by Grimme to correct for the dispersion interactions [29,34,35]. The kinetic cutoff energy was set to 450 eV to truncate the plane wave basis. The Brillouin zone was sampled using the Monkhorst–Pack scheme [36] with 3 × 3 × 1 k-meshes for structure relaxation. A 20 Å vacuum along the z-direction was utilized to prevent spurious interaction between the periodically repeated images. The convergence thresholds for energy and atomic force components were set to 10^−4^ eV and 0.05 eV/Å, respectively. It is worth mentioning that the obtained overpotentials can be very close to the corresponding ones from considering the solvent effect through VASPsol with a dielectric constant of 80, as revealed by our computational test on Ir@C_3_N_2_ (Figure S1). This indicates that the solvent effect can have a negligible influence. Therefore, to make the computational cost less demanding, in this study, we performed correlative calculations in a vacuum condition for estimating the OER catalytic activity for the studied systems. In addition, we also performed a DFT+U computational test on a 3d metal-doped *pc*-C_3_N_2_ system by sampling Co@C_3_N_2_ (Figure S2), where the Hubbard U parameters of Ueff = U − J = 3.1 eV (U = 4.0 eV and J = 0.9 eV) were adopted, according to previous work [37]. As shown in Figure S2, the OER overpotentials obtained by the DFT+U method can be comparable to the corresponding overpotentials calculated by DFT without U, indicating that all the present DFT results in this work can effectively evaluate the OER catalytic activity of relevant systems. 

To estimate the stability, the binding energy of TM@pc-C_3_N_2_ was calculated using the following formula:

E_b_ = E_pc-C_3_N_2__ + nE_TM_ − E_TM@pc-C_3_N_2__ (1)

where E_TM@pc-C_3_N_2__ and E_pc-C_3_N_2__ are the total energies of TM@pc-C_3_N_2_ and pristine pc-C_3_N, respectively; n is the number of doped transition metal atoms; E_TM_ is the energy of an isolated TM atom. 

The cohesive energy (E_c_) of a crystal is defined as the energy that must be added to the crystal to separate its components into neutral free atoms. The E_c_ value can be calculated by the following formula:

E_c_ = E_TM(bulk)_/N − E_TM_ (2)

where E_TM(bulk)_ is the total energy of a TM bulk, and N is the number of atoms in the bulk.

In electrochemistry, the overall OER in acidic solutions can usually be:

2H_2_O → O_2_ + 4H^+^ + 4e^−^ (3)

It can be divided into the following four elementary reaction steps [38]:

H_2_O + * → OH* + H^+^ + e^−^ (4a)

OH* → O* + H^+^ + e^−^ (4b)

H_2_O + O* → OOH* + H^+^ + e^−^ (4c)

OOH* → * + O_2_ + H^+^ + e^−^ (4d)

where * represents an active site on the catalyst surface, and OH*, O* and OOH* represent three different catalytic intermediates. 

Based on these reactions, the adsorption energies of these species can be obtained by the following expressions:

∆E_OH*_ = E_OH*_ − E_*_ − (E_H_2_O_ − 1/2E_H_2__) (5)

∆E_O*_ = E_O*_ − E_*_ − (E_H_2_O_ − E_H_2__) (6)

∆E_OOH*_ = E_OOH*_ − E_*_ − (2E_H_2_O_ − 3/2E_H_2__) (7)

where the E_*_, E_OH*_, E_O*_ and E_OOH*_ are the total energies of the catalyst substrate without and with the adsorption of OH, O or OOH, respectively; E_H_2_O_ and E_H_2__ are total energies of free H_2_O and H_2_ molecules in the gas phase, respectively.

The change in free energy ∆G can be obtained by the following expression:

∆G = ∆E + ∆ZPE − T∆S + ∆G_U_ + ∆G_pH_ (8)

where ∆E is the adsorption energy for the relevant intermediates involving OH, O and OOH. ∆ZPE and ∆S are the zero-point energy change and the change in entropy, respectively. ∆G_U_ = −eU, where U is the electrode potential related to the standard hydrogen electrode. ∆G_pH_ = k_B_TIn^10^ × pH is the correction for Gibbs free energy depending on the concentration of H+ ions, and pH = 0 was used in this study. The free energy G_(H+ + e-)_ is approximated with 1/2G_H_2__ for each elementary step involving the proton–electron pair. The free energy of O_2_ can obtained from the reaction 2H_2_O → O_2_ + 2H_2_, for which the free energy change is 4.92 eV.

Finally, the overpotential (η) of OER can be evaluated by the following formula:

η = max{∆G_a_, ∆G_b_, ∆G_c_, ∆G_d_}/e − 1.23 V (9)

## 3. Results and Discussion

### 3.1. Geometric Structure, Stability and Electronic Properties for TM@C_3_N_2_

**Figure 1:**
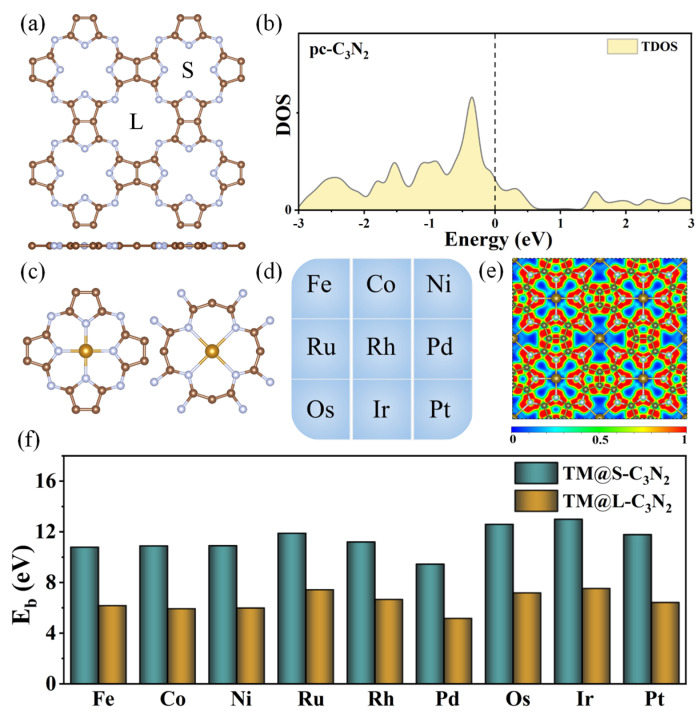
(**a**) Top view of the pc-C_3_N_2_ monolayer. (**b**) Density of states (DOS) of pc-C_3_N_2_. (**c**) Schematic diagram of doping transition metal (TM) into the small or large cavity. (**d**) The group VIII TM atoms considered in the current work. (**e**) The electron location function for TM@C_3_N_2_ systems. (**f**) Binding energy (E_b_) of TM atoms doped in the small (TM@S-C_3_N_2_) or large cavity (TM@L-C_3_N_2_).

A pristine pc-C_3_N_2_ monolayer belongs to the P4/MMM group, which can be considered an extended 2D network consisting of the cavities surrounded by four N atoms in pyrrole-like units or four N atoms connecting the pyrrole-like units (Figure 1a). This unique structure can make it act as a macrocyclic ligand to construct an SAC structure with TMN_4_ units by doping TM atoms. The optimized lattice constant of a unit cell for pc-C_3_N_2_ is a = b = 8.29 Å, which is consistent with previous results [27,28]. From Figure 1a, we can also find that there are two kinds of holes with different sizes in the pc-C_3_N_2_ monolayer, namely, one is the large hole surrounded by four N atoms connecting the pyrrole-like units (marked by L), and the other is the small hole surrounded by four N atoms from the pyrrole-like units (marked by S). Such holes with different sizes will be conducive to matching TM atoms with different atomic radii, and it is highly anticipated that the highly active centers of OER can be realized in the pc-C_3_N_2_ monolayer. In addition, the calculated density of states (DOS) results reveal that the pristine pc-C_3_N_2_ monolayer can exhibit the metallic characteristics (Figure 1b), indicating good conductivity. 

Subsequently, we investigated the geometric structures, stability and electronic properties of the doped pc-C_3_N_2_ systems, where the transition metal atoms in group VIII (TM = Fe, Co, Ni, Ru, Rh, Pd, Os, Ir and Pt) are used to embed into each large or small cavity (Figure 1c,d). For convenience, all these newly formed systems can be represented as TM@C_3_N_2_. After optimization, these TM@C_3_N_2_ systems can still maintain a planar structure with P4/MMM symmetry, where the TM atoms are located at the center of each hole, independent of the hole size. The calculated lattice parameters are in the range of 16.244~16.505 Å, which can be very close to that of the pristine pc-C_3_N_2_ (a = b = 16.580 Å), indicating a large structural rigidity. As shown in Table 1, the calculated TM-N bond lengths in the large cavity (2.294~2.409 Å) can be slightly longer than those in the small cavity (1.889~1.981 Å), but all of them can also be close to the corresponding TM-N bond length of experimentally obtained metal nitrides (1.830~2.300 Å). As a result, the TM atoms may be stably anchored in these cavities of the pc-C_3_N_2_ monolayer to form two different kinds of TMN_4_ units, which can be reflected by their large positive binding energies (E_b_) in the range of 5.17~13.00 eV, as shown in Figure 1e. Besides, it has been widely accepted that a ratio of binding energy to cohesive energy (−E_b_/E_c_) greater than 0.5 indicates that single atoms tend to separate on the substrate rather than aggregate together [39,40,41]. For all of these TM@C_3_N_2_ systems, the calculated −E_b_/E_c_ values can be greater than 0.5 (Table S1), and even most values are greater than 1, which means that TM atoms can be anchored at the holes individually rather than clustered together. 

**Figure 2:**
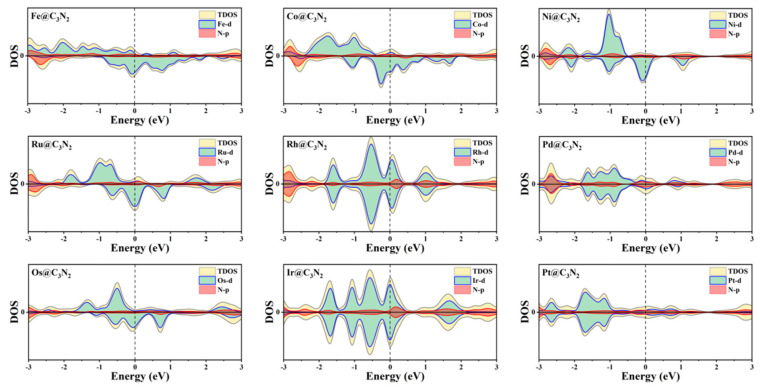
Density of states for TM@C_3_N_2_ systems.

Furthermore, the calculated electron location function (ELF) results reveal that all the TM-N bonds can exhibit the typical ionic bond characteristics, in view of the significant difference in ELF between the area around the TM (near to zero) and the N (about 0.9) atoms (Figure 1e). This can be further supported by the computed Bader charger analysis, where the electron of 0.58~1.23 |e| can be transferred from TM to its adjacent N atoms. In addition, we find that all the doped systems can maintain the metallic behavior (Figure 2) and that the DOS values from TM at the Fermi level can be larger than that of the pristine pc-C_3_N_2_, indicating that the conductivity is enhanced, which is conducive to the OER catalytic performance of the material.

### 3.2. OER Catalytic Activity of the TM@C_3_N_2_ Systems

The good stability and conductivity of these doped pc-C_3_N_2_ systems containing TMN4 units can promote us to explore their potential as SAC electrocatalysts for OER in the process of water splitting. Based on these TM@C_3_N_2_ structures, we systematically investigated their OER activity according to the scheme proposed by Rossmeisl et al. [38] In this method, the OER process is generally recommended to include four elementary reaction steps, each involving one proton/electron coupling transfer process, as shown in Formulas (4a)–(4d). The reaction overpotential (η) can be obtained by evaluating the difference between the minimum voltages required for the four reaction steps. Herein, we will explore the OER catalytic activity of TM@C_3_N_2_ systems by calculating their overpotential η values. By performing a computational screening of the group VIII elements (i.e., Fe, Co, Ni, Ru, Rh, Pd, Os, Ir and Pt) (Figure 1d), we find that four TM@C_3_N_2_ systems doped with Co, Rh, Ir and Ru atoms can exhibit very low overpotential in the range of 0.33~0.48 V, even smaller than the state-of-the-art IrO_2_ (0.56 V), indicating considerably high OER catalytic activity. Among them, a Rh@C_3_N_2_ system can display higher OER activity due to more active sites. 

Specifically, we have carried out calculations related to the OER process by adsorbing the OH, O and OOH species on the surface of TM@C_3_N_2_, where the TM atoms in small or large cavities are used as possible adsorption sites. For convenience, these different adsorption sites are labeled TM@S-C_3_N_2_ and TM@L-C_3_N_2_, respectively, according to the size of the embedded hole. 

**Figure 3:**
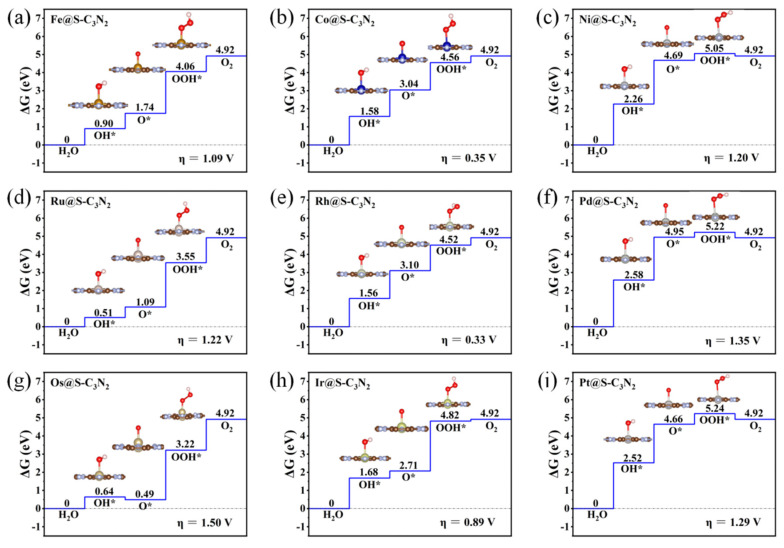
Gibbs free energy diagram of OER for the TM@S-C_3_N_2_ series including Fe@S-C_3_N_2_ (**a**), Co@S-C_3_N_2_ (**b**), Ni@S-C_3_N_2_ (**c**), Ru@S-C_3_N_2_ (**d**), Rh@S-C_3_N_2_ (**e**), Pd@S-C_3_N_2_ (**f**), Os@S-C_3_N_2_ (**g**), Ir@S-C_3_N_2_ (**h**) and Pt@S-C_3_N_2_ (**i**).

Subsequently, when doping 4d TM atoms (i.e., Ru, Rh and Pd) into the small cavity of pc-C_3_N_2_, a similar situation can be observed (Figure 3d–f). To be specific, with the increase in the outer electrons of the 4d TM atoms, the overpotential η of TM@S-C_3_N_2_ can also decrease sharply and then increase, and Rh@S-C_3_N_2_ (0.33 V) can exhibit much lower overpotential than Ru@S-C_3_N_2_ (1.22 V) and Pd@S-C_3_N_2_ (1.35 V). Obviously, high OER catalytic activity can also be achieved by doping Rh atoms with the same number of outer electron as Co atoms.

**Figure 4:**
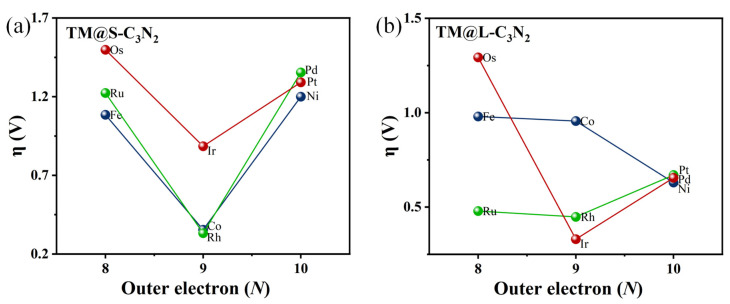
The relationships of η vs. outer electron (N) of TM for TM@S-C_3_N_2_ (**a**) and TM@L-C_3_N_2_ (**b**).

Similarly, doping a 5d transition metal Ir atom (0.89 V), which has the same number of outer electrons as Co and Rh atoms, can also induce lower overpotential than the parallel 5d TM atoms including Os (1.50 V) and Pt (1.29 V), as shown in Figure 3g–j. Clearly, when embedding the TM atoms of group VIII into the small cavity of pc-C_3_N_2_, the number of outer electrons can play an important role in determining the overpotential value of TM@S-C_3_N_2_. Employing TM atoms (Co, Rh and Ir) with nine outer electrons can produce a lower overpotential compared with other corresponding TM atoms in the same period (Figure 4). However, we can also find that the doping of Ir can lead to a relatively larger overpotential than Co and Rh in the same column, indicating that the periodic number of doped TM atoms also has an important influence on the overpotential, which can also be observed in the other two series (i.e., Fe/Rh/Os and Ni/Pd/Pt), as shown in Figure 4. Clearly, selecting a TM atom of group VIII in the appropriate period to match the hole size in pc-C_3_N_2_ can be advantageous for realizing the high OER catalytic activity. 

Overall, the number of outer electrons and the periodic number for the doped TM atoms can be considered two key factors to achieve high OER catalytic activity in 2D pc-C_3_N_2_, which can also be well reflected in the results of subsequently doping the group VIII TM atoms into the large cavity of pc-C_3_N_2_.

**Figure 5:**
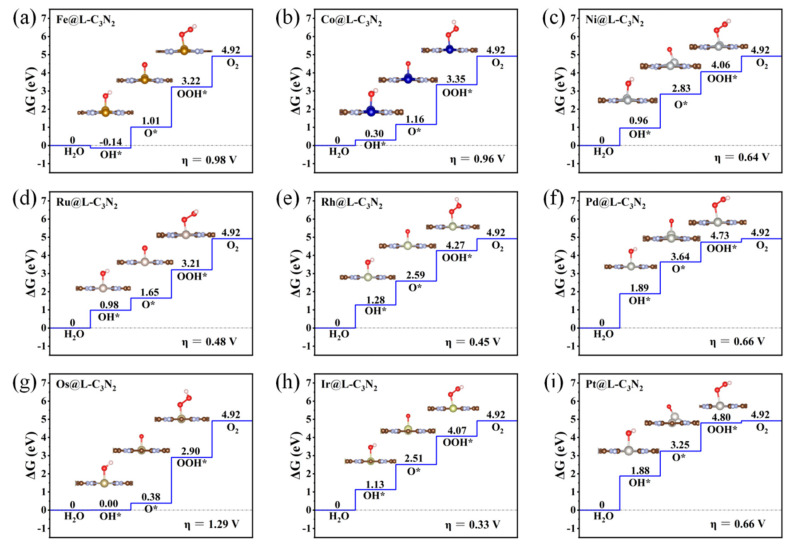
Gibbs free energy diagram of OER for TM@L-C_3_N_2_ series including Fe@L-C_3_N_2_ (**a**), Co@L-C_3_N_2_ (**b**), Ni@L-C_3_N_2_ (**c**), Ru@L-C_3_N_2_ (**d**), Rh@L-C_3_N_2_ (**e**), Pd@L-C_3_N_2_ (**f**), Os@L-C_3_N_2_ (**g**), Ir@L-C_3_N_2_ (**h**) and Pt@L-C_3_N_2_ (**i**).

Specifically, when embedding the 5d transition metal Ir atom in the high period into the large cavity of pc-C_3_N_2_, the overpotential of Ir@L-C_3_N_2_ can be significantly decreased to 0.33 V, far less than the corresponding Ir@S-C_3_N_2_ (0.89 V), indicating excellent OER catalytic activity (Figure 5). However, when doping 3d transition metal Co atom in the low period, the formed Co@L-C_3_N_2_ (0.96 V) displays much higher overpotential than Co@S-C_3_N_2_ (0.35 V), suggesting the inert OER activity (Figure 5). Obviously, the matching between the doped TM atoms and the pore size can have an important influence on the OER catalytic activity of these SAC systems with the rigid ligand. Indeed, when a 4d transition metal Rh atom in the middle period is introduced, it can not only match the small hole size in pc-C_3_N_2_ but also match the large hole size. As a result, Rh@S-C_3_N_2_ (0.33 V) and Rh@L-C_3_N_2_ (0.45 V) can uniformly exhibit very low overpotential (Figure 4 and Figure 5), reflecting considerably high OER catalytic activity. In view of the formation of more active sites, the Rh-doped pc-C_3_N_2_ system can exhibit the best OER catalytic performance in these doped systems with group VIII elements. In addition, different from the adsorption in the small cavity, the Ru atom anchored at the large cavity in pc-C_3_N_2_ can have very low overpotential (0.48 V), presenting high OER catalytic activity.

Obviously, doping group VIII atoms into a pc-C_3_N_2_ monolayer (for example, introducing Co/Rh into the small cavity or Ru/Rh/Ir into the large cavity) can be considered an effective strategy to realize high-efficiency SAC catalysts for OER. In particular, by embedding Rh atoms into a pc-C_3_N_2_ monolayer, higher OER catalytic performance can be achieved because more active sites are produced.

### 3.3. Mechanism Analysis of OER

**Figure 6:**
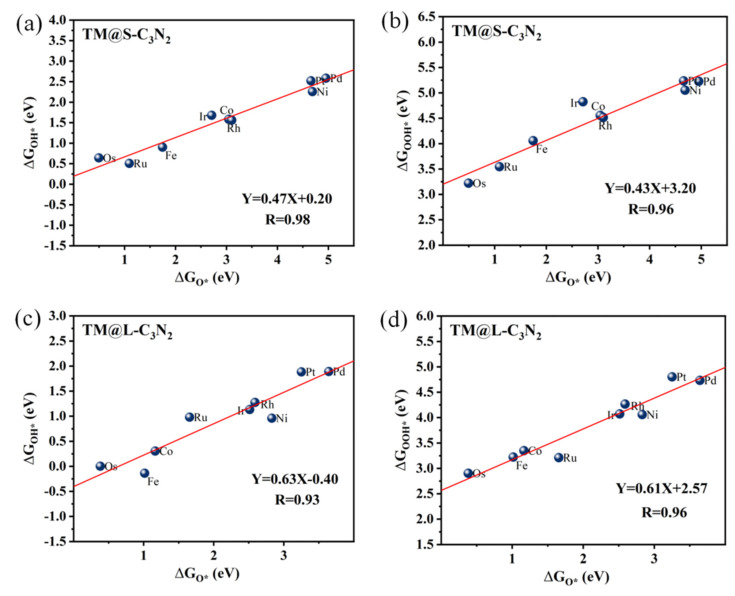
The scaling relationships corresponding to ∆G_O*_ vs. ∆G_OOH*_ and ∆G_O*_ vs. ∆G_OH*_ for TM@S-C_3_N_2_ (**a**,**b**) and TM@L-C_3_N_2_ (**c**,**d**).

**Figure 7:**
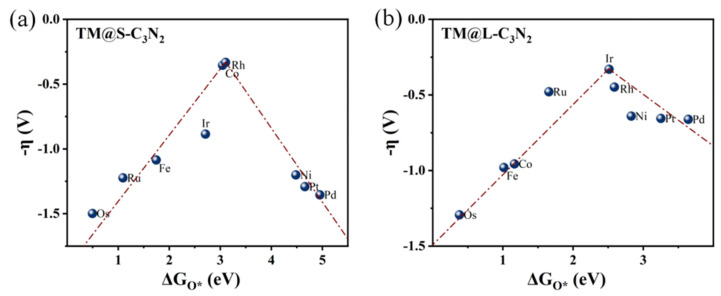
Volcano plots of -η vs. ∆G_O*_ for (**a**) TM@S-C_3_N_2_ and (**b**) TM@L-C_3_N_2_.

To understand the reasons behind the high OER catalytic activity of some TM@C_3_N_2_ systems, we further performed a detailed mechanism analysis. First, we clarified the scaling relationships of ∆G_O*_ vs. ∆G_OOH*_ and ∆G_O*_ vs. ∆G_OH*_ for the two adsorption sites corresponding to the TM atom located at the small (TM@S-C_3_N_2_) and large (TM@L-C_3_N_2_) cavities, respectively. From Figure 6, it is found that both the ∆G_OH*_ and ∆G_OOH*_ values can be linearly proportional to ∆G_O*_ uniformly, regardless of the size of the emmbeded hole in pc-C_3_N_2_. These good linear relationships can be expressed as ∆G_OH*_ = 0.47∆G_O*_ + 0.20 (correlation coefficients R = 0.98) and ∆G_OOH*_ = 0.43∆G_O_* + 3.20 (R = 0.96) for the TM@S-C_3_N_2_ site and ∆G_OH*_ = 0.63∆G_O*_ − 0.40 (R = 0.93) and ∆G_OOH*_ = 0.61ΔG_O*_ + 2.57 (R = 0.96) for the TM@L-C_3_N_2_ site, respectively. Therefore, ∆G_O*_ can be used as a valid descriptor related to the overpotential value (η), which can be also reflected by the volcano curve between ∆G_O*_ and η, as shown in Figure 7. 

Initially, we focus on TM@S-C_3_N_2_ systems in which group VIII atoms are doped into the small cavity. Specifically, we can find from Figure 7a that, when Fe, Ru and Os atoms doped in the small hole are used as adsorption sites (TM@S-C_3_N_2_), they can be located on the left side of the volcano curve, indicating that there is a strong interaction between O* and these TM atoms with eight outer electrons. In contrast, when the doped Ni, Pd and Pt atoms with ten outer electrons act as adsorption centers, they can be situated at the right side of the volcano curve, suggesting a weak interaction between O* and these TM sites. It is known that in general, the adsorption strength of reaction intermediates (such as O*) is crucial to determine the OER activity of the catalyst. Too strong or too weak adsorption of the intermediates is unfavorable to the occurrence of a catalytic reaction, in view of the fact that the former will block the catalytic site while the latter cannot provide enough driving force to bind adsorbates [42]. Therefore, when doping the Co/Rh/Ir atoms with nine outer electrons in the middle, high OER catalytic activity can be observed, because they can bring the appropriate adsorption strength of O*, which can be reflected by the fact that they are generally at the peak of the volcano curve.

**Figure 8:**
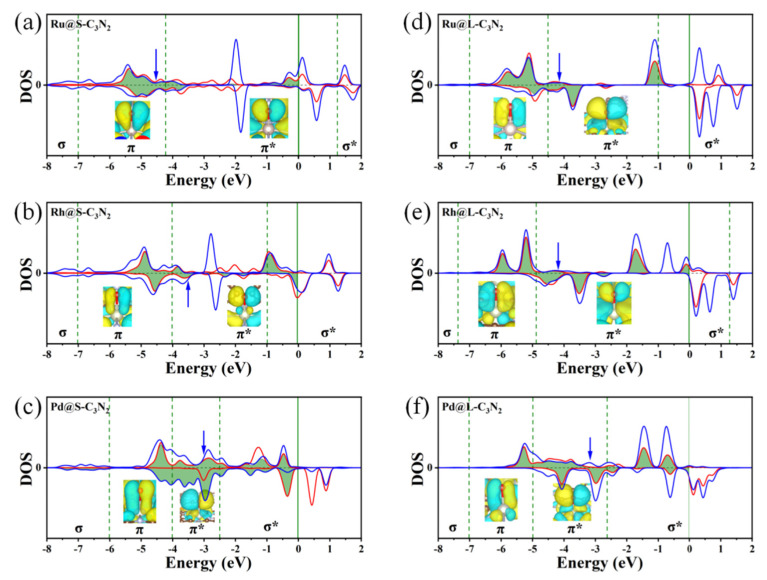
Partial density of states (PDOS) of the *p* orbitals of O atoms and the *d* orbitals of TM atoms after the O* adsorption of Ru@S-C_3_N_2_ (**a**), Rh@S-C_3_N_2_ (**b**), Pd@S-C_3_N_2_ (**c**), Ru@L-C_3_N_2_ (**d**), Rh@L-C_3_N_2_ (**e**) and Pd@L-C_3_N_2_ (**f**). Inset: the molecular orbitals of O atom adsorbed at the TM-site in different energy ranges marked by green dashed lines. Blue arrow represents the *p*–*d* overlap center.

To better understand that doping these TM atoms with nine outer electrons can induce the appropriate O* adsorption state, we conduct a deep bonding analysis of the intercation between the O* and the TM center by sampling the representive Rh@S-C_3_N_2_. For a parallel comparison, the relevant bonding analysis on the O* adsorption of doped Ru and Pd metal centers (located on the left and right sides of the volcano curve, respectively) in the same period is also considered. As shown in Figure 8a, when the Ru with eight outer electrons serves as the adsorption site, the center of overlapping O-p and Ru-d orbitals is located in the π bonding area for Ru@S-C_3_N_2_, resulting in a strong interaction between the O* and the TM center. Comparatively, when a Rh atom is introduced into the small cavity, the filling of the outer electron will increase, and the overlapping center of the O-p and Rh-d orbitals can enter the π* antibonding area. The appearance of an antibonding characteristic will effectively weaken the interaction strength between the O* and the TM center, bringing about an appropriate adsorption state of the O* (Figure 8b). Further, when doping Pd with more outer electrons, the overlapping center of the O-p and Pd-d orbitals in the π* antibonding area can continue to move towards the Fermi level, indicating an increase in antibonding characteristics (Figure 8c), which will lead to too-weak interaction between the O* and the TM, as reflected by the large positive ∆G_O*_ value.

Clearly, the filling of outer electrons can play a key role in determining the high OER catalytic activity of TM@S-C_3_N_2_ sites by modulating the ∆G_O*_ value. When doping these TM atoms into the large cavity in pc-C_3_N_2_ (TM@L-C_3_N_2_), a similar situation can also be observed, that is, as the outer electron number of the TM center increases, their positions can usually change from the left side of the volcano curve to the peak and then to the right side (Figure 7b). This gradual weakening of the interaction between the O* and the TM center can also be reasonably explained by sampling the bonding analysis of the representative Ru/Rh/Pd-doped systems. As shown in Figure 8d,f, we can find that the overlapping center of the O-p and TM-d orbitals can be located in the π* bonding region and gradually approaches the Fermi level with the increase in the outer electrons, which leads to the enhancement of antibonding characteristics and the weakening of the interaction between the O* and the TM site. In addtion, unlike the case of the corresponing small cavity, when a Ru atom is doped into the large cavity in pc-C_3_N_2_, the overlapping center of the O-p and Ru-d orbitals has been located in the π* bonding region (Figure 8d), which can provide an appropriate O* adsorption state and induce high OER catalytic activity.

**Figure 9:**
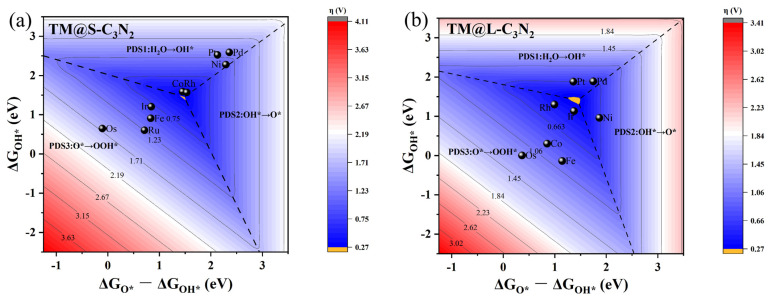
The 2D colored contour plots of OER activity volcanos for (**a**) TM@S−C_3_N_2_ and (**b**) TM@L−C_3_N_2_ by presenting the η values as a function of the Gibbs free energies.

Finally, the limiting overpotential η values of the TM@S-C_3_N_2_ and TM@L-C_3_N_2_ sites are evaluated by constructing the two-dimensional volcano diagram according to the linear relationships from Figure 6, in which the change of free energy (∆G) in each step of OER can be directly related to ΔG_O*_. This method has been successfully applied in previous works [42,43,44]. Figure 9 shows that the volcano diagrams can be divided into three regions, which correspond to the different potential determining steps (PDSs). It can be found that the adsorption strength of O* can effectively affect the PDS step of the OER reaction. The samples with relatively weak O* adsorption are generally located in the PDS1 (H_2_O → OH*) or PDS2 (OH* → O*) area, while those with strong O* adsorption are located in the PDS3 (O* → OOH*) area. Herein, the yellow regions in the two volcano diagrams predict the limiting overpotentials η of the TM@S-C_3_N_2_ and TM@L-C_3_N_2_ series, both of which can be as small as 0.27 V. It is worth mentioning that, in the systems we designed, the overpotentials of some active sites (such as Co@S-C_3_N_2_, Rh@S-C_3_N_2_ and Ir@L-C_3_N_2_) can be as low as 0.33–0.35 V, very close to the limit value. All these findings can provide very in depth insight into the design of SAC catalysts with high OER activity under the rigid pc-C_3_N_2_ framework.

## 4. Conclusions

In this study, we constructed a series of two-dimensional SAC systems by doping the TM atoms of the group VIII into the cavities of a rigid pc-C_3_N_2_ monolayer and systematically investigate their structures and OER catalytic performance based on the DFT calculations. The following intriguing findings can be obtained:

(1) All these TM@C_3_N_2_ systems can exhibit high structural stability, wherein the TM atoms are stably anchored in small and large cavities of a pc-C_3_N_2_ monolayer to form two different kinds of TMN_4_ units. Compared with the pristine pc-C_3_N_2_ with metallic characteristics, the conductivity of these doped systems can be further enhanced. All these advantages are conducive to the OER catalytic performance of the materials.
(2) Through a calculation screening of the TM atoms in group VIII, it is found that four new TM@C_3_N_2_ systems doped with Co/Rh/Ir/Ru atoms can possess very low overpotential (0.33~0.48 V), indicating the considerably high OER catalytic activity, where the adsorption sites including Co@S-C_3_N_2_, Rh@S-C_3_N_2_, Ru@L-C_3_N_2_, Rh@L-C_3_N_2_ and Ir@L-C_3_N_2_ can be used as the active sites. As a result, the Rh@C_3_N_2_ system can exhibit higher OER catalytic performance, due to the higher density of active sites.
(3) It is found that ∆G_O*_ can be used as an effective descriptor of the OER catalytic activity of TM@C_3_N_2_ systems. The number of outer electrons, the periodic number of doped TM atoms and the cavity size can be the crucial factors in determining the ∆G_O*_ value, and the effective cooperation between them can lead to moderate ∆G_O*_ values, bringing about excellent OER catalytic performance in these SAC catalysts.

Obviously, introducing group VIII atoms with nine outer electrons into the cavities of a rigid pc-C_3_N_2_ ligand can be considered an effective strategy to realize SAC catalysts with high OER catalytic performance. All these fascinating findings are conducive to the design of new and efficient OER catalysts.

## Figures and Tables

**Table 1:** The calculated lattice parameters of pc-C_3_N_2_ and TM@C_3_N_2_ and the calculated TM-N bond lengths for TM@C_3_N_2_.

	a = b (Å)	D_S-TM-N_ (Å)	D_L-TM-N_ (Å)
pc-C_3_N_2_	16.580	---	---
Fe@C_3_N_2_	16.345	1.914	2.356
Co@C_3_N_2_	16.385	1.952	2.333
Ni@C_3_N_2_	16.234	1.889	2.321
Ru@C_3_N_2_	16.261	1.889	2.331
Rh@C_3_N_2_	16.370	1.951	2.342
Pd@C_3_N_2_	16.300	1.942	2.294
Os@C_3_N_2_	16.244	1.891	2.323
Ir@C_3_N_2_	16.505	1.981	2.409
Pt@C_3_N_2_	16.400	1.971	2.350

## Data Availability

Not applicable.

## Supplementary Materials

The following supporting information can be downloaded at: https://www.mdpi.com/article/10.3390/molecules28010254/s1, Figure S1: Gibbs free energy diagram of OER in solvation condition for Ir@S-C_3_N_2_(a) and Ir@L-C_3_N_2_(b); Figure S2: Gibbs free energy diagram of OER for Co@C_3_N_2_ under the DFT+U method; Table S1: The binding energy E_b_ (eV), cohesive energy E_c_ (eV), and the ratio of −E_b_/E_c_ for the studied systems.

## Sample Availability

Samples are available from the authors.
