# Supplementary material for: Embedding Group VIII Elements into a 2D Rigid pc-C3N2 Monolayer to Achieve Single-Atom Catalysts with Excellent OER Activity: A DFT Theoretical Study
Source: Molecules. 2022 Dec 28;28(1):254. doi: 10.3390/molecules28010254 (PMC9821954; doi:10.3390/molecules28010254)
Supplement: Supplementary file 1 [file molecules-28-00254-s001.zip › molecules-2066096-supplementary.pdf]

Supporting Information

Embedding VIII group Elements into 2D Rigid pc-C_3N_2 Monolayer to Achieve the Single-Atom Catalysts with Excellent OER Activity: a DFT Theoretical Study

Qingxian Wang [1], E Yang[1], Ran Liu [1], Mingyue Lv[1], Wei Zhang[2,3,*], Guangtao Yu [1,*] and Wei Chen[1,2,4,*]

[1] Engineering Research Center of Industrial Biocatalysis, Fujian Provincial Key Laboratory of Advanced Materials Oriented Chemical Engineering, Fujian-Taiwan Science and Technology Cooperation Base of Biomedical Materials and Tissue Engineering, College of Chemistry and Materials Science, Fujian Normal University, Fuzhou 350007, China

[2] Academy of Carbon Neutrality of Fujian Normal University, Fuzhou 350007, China

[3] Fujian Provincial Key Laboratory of Quantum Manipulation and New Energy Materials, College of Physics and Energy, Fujian Normal University, Fuzhou 350117, China

[4] Fujian Provincial Key Laboratory of Theoretical and Computational Chemistry, Xiamen University, Xiamen 361005, China

* Corresponding authors: dress: zhangw721@163.com (W. Zhang); yugt@fjnu.edu.cn (G. Yu); chenwei@fjnu.edu.cn (W. Chen)

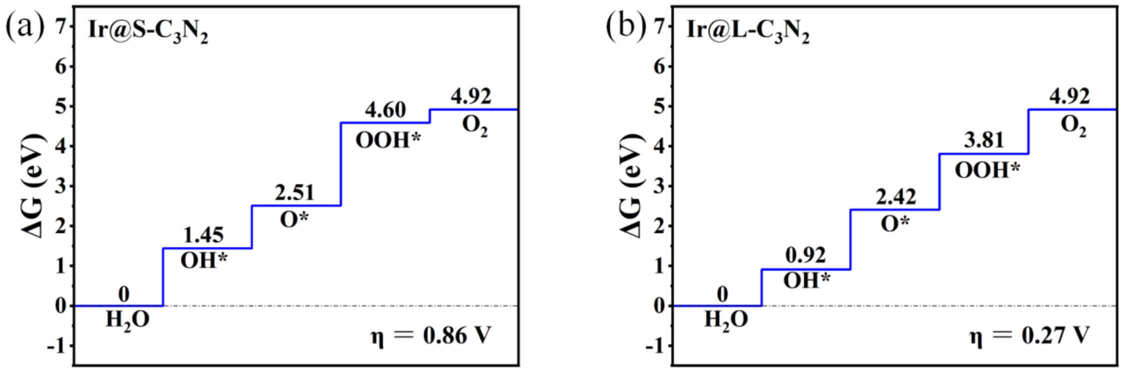

Figure S1. Gibbs free energy diagram of OER in solvation condition for Ir@C_3N_2.

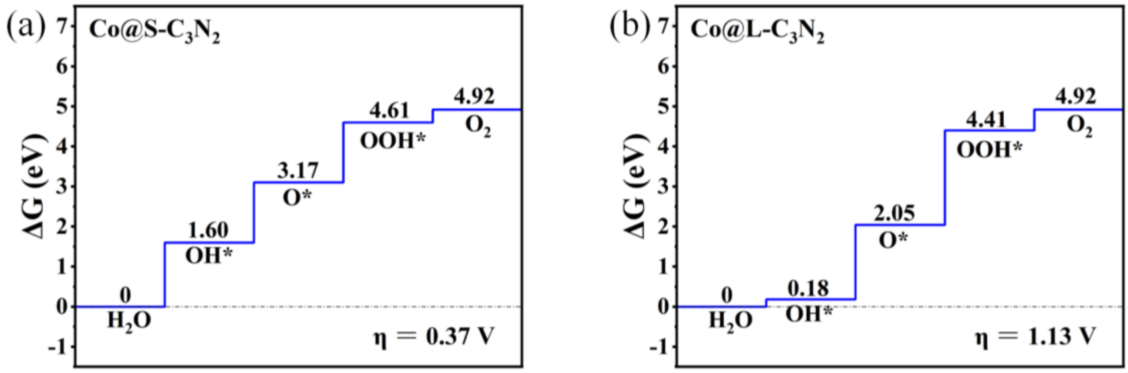

Figure S2. Gibbs free energy diagram of OER for Co@C_3N_2 under the DFT+U method.

Table S1. The binding energy E_b (eV), cohesive energy E_c (eV), and the ratio of $-E_b/E_c$ for the studied systems.

	E_b	E_c	$-E_b/E_c$
Fe@S-C_3N_2	10.79	-5.37	2.01
Co@S-C_3N_2	10.89	-8.06	1.35
Ni@S-C_3N_2	10.90	-8.37	1.30
Ru@S-C_3N_2	11.89	-5.62	2.12
Rh@S-C_3N_2	11.20	-6.54	1.71
Pd@S-C_3N_2	9.45	-7.62	1.24
Os@S-C_3N_2	12.59	-5.41	2.33
Ir@S-C_3N_2	12.99	-4.32	3.01
Pt@S-C_3N_2	11.78	-5.55	2.12
Fe@L-C_3N_2	6.18	-5.37	1.15
Co@L-C_3N_2	5.93	-8.06	0.74
Ni@L-C_3N_2	5.99	-8.37	0.72
Ru@L-C_3N_2	7.43	-5.62	1.32
Rh@L-C_3N_2	6.66	-6.54	1.02
Pd@L-C_3N_2	5.17	-7.62	0.68
Os@L-C_3N_2	7.18	-5.41	1.33
Ir@L-C_3N_2	7.53	-4.32	1.74
Pt@L-C_3N_2	6.42	-5.55	1.16
